# Steroid treatment for the first episode of childhood nephrotic syndrome: comparison of the 8 and 12 weeks regimen using an individual patient data meta-analysis

**DOI:** 10.1007/s00431-021-04035-w

**Published:** 2021-03-28

**Authors:** Anne M. Schijvens, Nynke Teeninga, Eiske M. Dorresteijn, Steven Teerenstra, Nicholas J. Webb, Michiel F. Schreuder

**Affiliations:** 1 Department of Pediatric Nephrology, Radboud University Medical Center, Radboud Institute for Molecular Life Sciences, Amalia Children’s Hospital, Nijmegen, the Netherlands; 2 Department of Pediatric Nephrology, Erasmus University Medical Center, Sophia Children’s Hospital, Rotterdam, the Netherlands; 3 Department for Health Evidence, section Biostatistics, Radboud Institute for Health Sciences, Radboud University Medical Center, Nijmegen, the Netherlands; 4 Department of Paediatric Nephrology, Royal Manchester Children’s Hospital, Manchester, UK; 5 Manchester Academic Health Science Centre, University of Manchester, Manchester, M13 9PL UK

**Keywords:** Nephrotic syndrome, IPD meta-analysis, Prednisolone, Children

## Abstract

Steroids are the cornerstone of the treatment of childhood nephrotic syndrome. The optimal duration for the first episode remains a matter of debate. The aim of this study is to determine whether the 8 weeks International Study of Kidney Disease in Children (ISKDC) regimen is equally effective as the 12 weeks steroid regimen from the German society of pediatric nephrology (Arbeitsgemeinschaft für Pädiatrische Nephrologie [APN]). An individual patient data (IPD) meta-analysis of randomized controlled trials reporting on prednisolone treatment for a first episode of childhood nephrotic syndrome was conducted. European trials aimed at investigating the ISKDC and/or APN steroid regimen were selected. The lead investigators of the selected trials were requested to provide the IPD of the specific treatment groups. Four trials included European cohorts using dosing schedules according to the regimens studied. IPD of two trials were available. A significant difference was found in time to first relapse after cessation of steroid treatment between the 8 and 12 weeks treatment group with a median time to relapse of 29 and 63 days, respectively. Moreover, relapse rate ratios during total follow-up were 51% higher for the 8 weeks regimen. Finally, younger children have a significantly lower time to first relapse and frequently relapsing nephrotic syndrome.

*Conclusions*: The results of this IPD meta-analysis suggest that the 8 weeks steroid regimen for a first episode of steroid-sensitive nephrotic syndrome may not be equally effective as the 12 weeks steroid regimen. Moreover, this study highlights the importance of using uniform definitions to enable accurate comparison and interpretation of trial results.

*Trial registration*: Registration number: CRD42020199244, date of registration 16-08-2020

**Table:** 

**What is Known:** *• Steroids are the cornerstone of the treatment of childhood nephrotic syndrome, however the optimal duration for the first episode remains a matter of debate.* *• Currently, the 8 weeks ISKDC protocol and 12 weeks APN protocol are among the most frequently used protocols in Europe.*	
**What is New:** *• The 8 weeks steroid regimen for a first episode of steroid-sensitive nephrotic syndrome may not be equally effective as the 12 weeks steroid regimen for the treatment of a first episode of nephrotic syndrome.* *• Younger children have a significantly shorter time to first relapse and time to frequent relapsing nephrotic syndrome.*

**Supplementary Information:**

The online version contains supplementary material available at 10.1007/s00431-021-04035-w.

## Introduction

Nephrotic syndrome is one of the most common glomerular disorders in childhood with a reported incidence of 1–2 per 100,000 children per year [1–4]. For over 60 years, steroids have been the cornerstone of the treatment of nephrotic syndrome as over 80–90% of patients achieve complete remission after steroid treatment [5, 6]. Current recommendations on the treatment of a first episode of nephrotic syndrome are based on empirical experience and small randomized controlled trials. The 2012 Kidney Disease: Improving Global Outcomes (KDIGO) Clinical Practice Guideline for Glomerulonephritis recommends that corticosteroid therapy should be given for at least 12 weeks [7]. Moreover, it is stated that daily oral prednisone is given for 4–6 weeks followed by alternate day medication for 2–5 months with tapering of the dose. This recommendation leaves room for interpretation. In fact, wide variability is present among European centers in the treatment of a first episode of nephrotic syndrome [8]. Recent, well-conducted trials clearly show no benefit of steroid therapy beyond 12 weeks; however they are unable to provide information on the preference for an 8 or 12 weeks steroid regimen [9–12]. Consequently, the 8 weeks International Study of Kidney Disease in Children (ISKDC) protocol and 12 weeks Arbeitsgemeinschaft für Pädiatrische Nephrologie (APN) protocol are among the most frequently used protocols in Europe [8]. Recently, a Cochrane review suggested little or no difference between the 8 and 12 weeks steroid regimens [13]. However, a direct comparison between these commonly used regimens has only been made in small cohorts and the results seem contradictory [14–17]. The aim of this study is to determine whether the 8 weeks steroid regimen for a first episode of nephrotic syndrome in European children is equally effective as the 12 weeks steroid regimen using an individual patient data (IPD) meta-analysis.

## Materials and methods

### Protocol and registration

Methods were pre-specified in a protocol that was registered in PROSPERO (CRD42020199244).

### Paper and patient selection

We conducted an IPD meta-analysis of randomized controlled trials reporting on steroid treatment for a first episode of childhood nephrotic syndrome. This IPD meta-analysis was reported according to the Preferred Reporting Items for Systematic Review and Meta-Analyses of Individual Participant Data (PRISMA-IPD) statement [18]. We identified trials from the Cochrane reviews of Hahn et al. [13, 19]. Additionally, a PubMed search was conducted (March 2, 2020) to identify additional trial reports. In brief, the search strategy included the following terms: (“Nephrotic syndrome”[Mesh] OR nephrotic syndrome [tiab] OR nephrotic OR nephrosis [tiab]) AND (Infan* OR kid OR kids OR child OR child* OR children* OR schoolchild* OR schoolchild OR school child[tiab] OR school child*[tiab] OR adolescen* OR juvenil* OR youth* OR teen* OR under*age* OR pubescen* OR pediatrics[mh] OR pediatric* OR paediatric* OR peadiatric*) AND (steroids OR corticosteroids OR prednisolone OR prednisone) AND (trial OR clinical trial).

### Eligibility criteria

We selected randomized controlled trials recruiting pediatric nephrotic syndrome patients treated according to the ISKDC protocol and/or the APN protocol. From the selected trials, only children treated according to the aforementioned protocols were included in the analysis. Only European trials were included as ethnicity is considered to play a role in susceptibility to the disease as well as in the responsiveness to steroids [20, 21]. As this resulted in a low number of cohorts that could be included, US and Canadian studies were added as well, despite the potential higher impact of ethnicity on the outcome. Unfortunately, this did not yield any additional cohorts, and we therefore returned to our predefined criteria.

### Trial selection

Titles and abstracts were reviewed to exclude irrelevant studies, and full-text articles were evaluated for their eligibility by three members of the study group. The lead investigators of the selected trials were requested to provide IPD. When IPD were not available, aggregate results of trials directly comparing the two treatment regimens were compared to the results of the IPD meta-analysis.

### Data collection

The data requested for each patient included sex, ethnicity, age at diagnosis, treatment protocol, weight at presentation, height at presentation, relapse (yes/no), time to relapse, frequently relapsing nephrotic syndrome (FRNS) (yes/no), time to FRNS, steroid-dependent nephrotic syndrome (SDNS) (yes/no), time to SDNS, number of relapses during 12-month follow-up, number of relapses during 24-month follow-up, and the start of immunosuppressive maintenance therapy. We used standard checks to identify missing data, assess data validity, and consistency. Sex, ethnicity, and age at diagnosis were considered as covariates. Ethnicity was classified as Caucasian, Asian, or others. Age at diagnosis was stratified as < 4 years and ≥ 4 years as it is suggested that younger children have a higher risk of a complicated disease course [10, 12, 22]. Definitions of FRNS and SDNS varied significantly among the trials (Supplemental Table 1). The definitions were adjusted to enable comparison of trial results in this IPD meta-analysis (Supplemental Table 1). Data were collected in IBM SPSS statistics version 25.

### Study objectives

The objective of this study was to determine whether the ISKDC steroid regimen (4 weeks of daily steroids 60 mg/m^2^, 4 weeks of alternate day steroids 40 mg/m^2^) for a first episode of steroid-sensitive nephrotic syndrome in children is equally effective as the steroid regimen proposed by the APN (6 weeks of daily steroids 60 mg/m^2^, 6 weeks of alternate day steroids 40 mg/m^2^). The primary outcome was defined as the time from the final prednisolone dose to the first relapse. As the median follow-up duration differed between the trials, the minimum follow-up duration across all selected trials was used, which was 24 months. Secondary outcomes included the progression to FRNS, SDNS; the number of relapses during 12, 24 months, and final follow-up; and the start of immunosuppressive maintenance therapy. In case time to SDNS was not recorded in the trial, progression to SDNS was analyzed for the full follow-up duration, and time to SDNS was excluded from the analysis.

### Statistical methods

The primary outcome was analyzed using Kaplan-Meier survival curves to visually present time to first relapse. We compared the time to events using a log rank test. Hazard ratios (HR) and 95% confidence intervals (CI) were calculated using a Cox proportional hazard model. Time to FRNS was analyzed similarly. All survival analyses were carried out using the time after cessation of steroid therapy to avoid bias in favor of the extended steroid course group. Categorical outcomes were analyzed using a Chi-square test, and relative risks (RRs) compared to the reference category were calculated. For dichotomous outcomes, RRs and 95% CI were used. Poisson regression and negative binomial regression in combination with follow-up time as an offset variable were used to calculate relapse rate ratios (RRR). An important methodological issue in IPD meta-analyses is the choice between the one-stage and two-stage approach. As in our analysis, only one trial had an arm with the 8 weeks treatment and one (others) trial had an arm with the 12 weeks treatment, we extracted the IPD of those treatment arms, and we conducted a two-group comparison. As patients within the 8 weeks were from one trial and the 12 weeks treatment patients were from another trial, it was not possible to estimate trial-effects, and we had to consider both groups as separate cohorts. Therefore, we compared baseline characteristics to assess confounding. Additionally, all analyses were corrected for the covariates gender, age, and ethnicity. Finally, there was one trial directly comparing the two treatment regimens but with no IPD. The aggregate results of that trial were compared with the results of the IPD meta-analysis to assess consistency. For each outcome, the odds ratios (OR) of that trial were compared to the odds ratio obtained in the IPD using a logistic regression analysis with the main effects and interaction term for treatment regimen (8 versus 12 weeks) and data source (the trial versus the IPD). Time to event data could not be compared as ranges or standard deviations were not reported in the manuscript. All analyses were carried out using IBM SPSS statistics version 25.

## Results

### Paper and patient selection (Fig. 1)

**Fig. 1:**
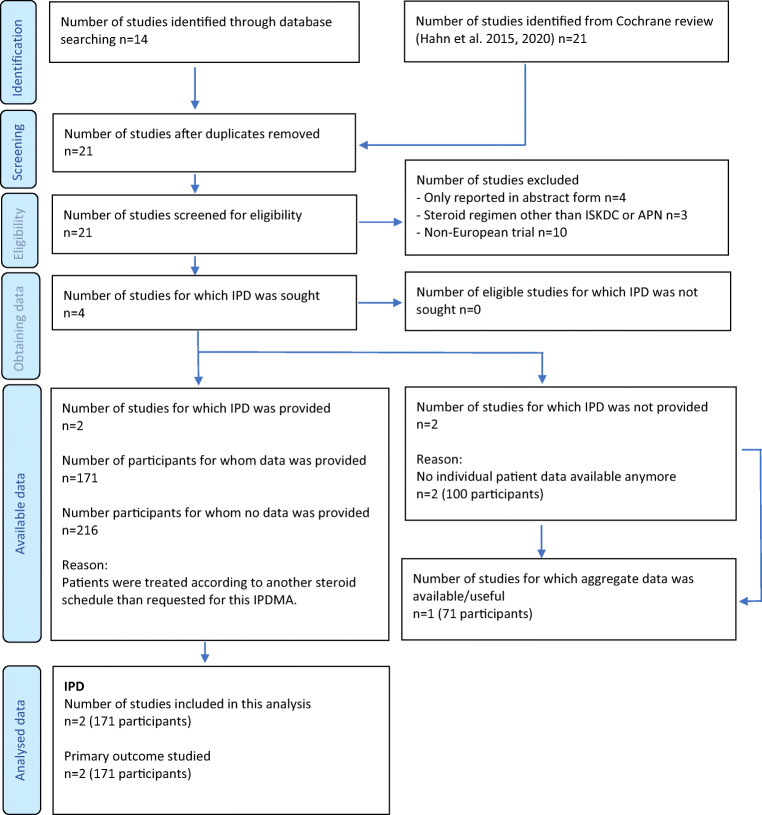
PRISMA individual patient data flow-diagram

The final search yielded 21 non-duplicated studies (Supplemental Table 2). Four studies were excluded as they only reported the results in abstract form and no full results were available [Jayantha UK, 2004, CN-00583710; Pecoraro C, 2005, CN-00644161; Satomura K 2001, CN-00447593; Sharma RK 2000, CN-00550434]. Three trials were excluded as other steroid regimens or cointerventions were used [23–25]. Finally, 10 non-European trials were excluded, none of which were US or Canadian studies [11, 12, 14–16, 22, 26–29]. The remaining four papers were included as they included European patient cohorts using the mg/m^2^ dosing schedule for either 8 weeks or 12 weeks or both [9, 10, 17, 30]. The primary investigators were contacted to share IPD and all investigators responded to our request. The trials of Webb et al. and Teeninga et al. provided IPD and data from the “short treatment arms” were included in this analysis (Fig. 1) [9, 10]. The APN studies of 1988 and 1993 [17, 30] had no IPD available anymore. As the Ehrich trial provided a direct comparison between the 8- and 12 weeks treatment, it was included in a separate analysis [17]. Study characteristics of the included trials are shown in Supplemental Table 3.

### Participants

In total, 171 patients aged 1.5–16 years were included in the IPD meta-analysis. As shown in Table 1, baseline characteristics did not significantly differ between the 8 and 12 weeks treatment groups, with the exception of ethnicity. In the original trials, children with steroid resistant nephrotic syndrome were excluded, which was defined as no clinical response after 4 (Webb) or 6 (Teeninga) weeks of daily prednisolone at 60 mg/m^2^/day.

**Table 1:** Baseline characteristics of the IPD data

	8 weeks protocol[10]	12 weeks protocol[9]	*p* Value
Number of patients	109	62	
Median (IQR) age at presentation (months)	54 (34.5–82.0)	57 (38.8–69.3)	0.73
Age category			
Age < 4 years Age ≥ 4 years	47 (43%) 62 (57%)	26 (42%) 36 (58%)	0.88
Gender			
Male Female	78 (72%) 31 (28%)	39 (63%) 23 (37%)	0.24
Ethnicity Caucasian Asian Others	(*n* = 108) 73 (68%) 25 (23%) 10 (9%)	(*n* = 55) 46 (83.5%) 2 (3.5%) 7 (13%)	0.01
Median (IQR) height at presentation (cm)	104 (92.2–122.0)	109.5 (98.8–118.3)	0.29
Median (IQR) weight at presentation (kg)	19.4 (15.2–24.4)	20.2 (17.0–25.4)	0.18
Total prednisolone dose first treatment (mg/m^2^)	2240	3360	
Median (IQR) follow-up duration study participants (months)	37 (30–48)	46 (32–60)	

*IQR* interquartile range

### Primary outcome

**Fig. 2:**
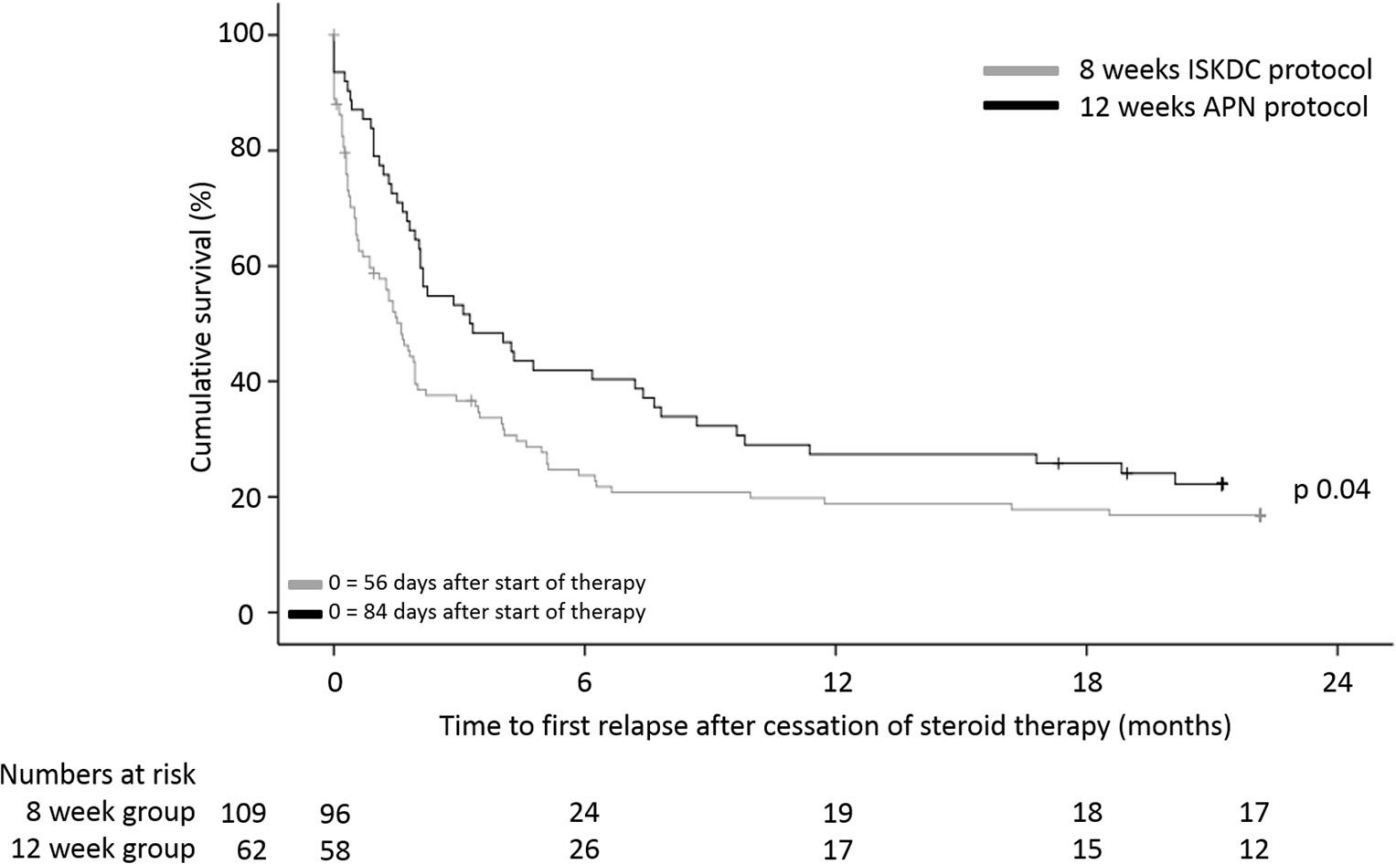
Time to first relapse after cessation of prednisolone therapy. APN, Arbeitsgemeinschaft für Pädiatrische Nephrology; ISKDC, International Study of Kidney Disease in Children

The proportion of patients having a relapse within 24 months of follow-up was 80% and 77% in the 8 weeks and 12 weeks group, respectively (RR = 1.03 [0.88-1.22]). The median time to relapse of participants experiencing a relapse was 29 days for the 8 weeks treatment group and 63 days for the 12 weeks treatment group (log rank, *p* = 0.04, Table 2, Fig. 2), calculated from the end of the steroid treatment. Cox proportional hazards regression analysis suggests children below 4 years of age to have a significantly shorter time to first relapse compared to children of 4 years and older (HR = 0.65 [0.45–0.93], Table 3).

**Table 2:** Primary and secondary outcomes at 24 months after comparison of the 8 and 12 weeks treatment regimens from IPD data

	8 weeks treatment group	12 weeks treatment group	Risks
Relapse	87 (80%)	48 (77%)	RR (95% CI) 1.03 (0.88–1.22)
Sustained remission*	21 (19%)	14 (23%)	RR (95% CI) 0.85 (0.47–1.56)
SDNS**	48 (44%)	30 (48%)	RR (95% CI) 0.91 (0.65–1.27)
FRNS**	52 (48%)	26 (42%)	RR (95% CI) 1.14 (0.80–1.62)
Start of immunosuppressive maintenance therapy***	61 (56%)	28 (45%)	RR (95% CI) 1.24 (0.90–1.71)

*FRNS* frequent relapsing nephrotic syndrome, *HR* hazard ratio, *IQR* interquartile range, *KDIGO* Kidney Disease: Improving Global Outcomes, *N/A* not available, *RR* relative risk, *SDNS* steroid-dependent nephrotic syndrome

RR is reported with the 12 weeks treatment group as a reference group

*Sustained remission at total follow-up; see Supplemental Table 3 for median (IQR) follow-up duration

**Definition 3 that enables comparison of trial results (Supplemental Table 1)

***For the patients of the Webb trial, only the start of second-line immunosuppressive maintenance therapy was recorded, whereas for the patients of the Teeninga trial, the start of any additional immunosuppressive therapy was recorded, including maintenance therapy with steroids

**Table 3:** Multivariate logistic regression analysis and cox proportional hazards analysis on outcome at 24 months adjusted for treatment, age, gender, and ethnicity

	Relapse	FRNS	SDNS	Time to first relapse	Time to FRNS
Covariate	OR (95% CI)	OR (95% CI)	OR (95% CI)	HR (95% CI)	HR (95% CI)
Treatment					
8 weeks 12 weeks (reference)	0.73 (0.32–1.67)	0.70 (0.35–1.40)	1.14 (0.58–2.25)	0.63 (0.42–0.93)	0.58 (0.34–0.98)
Gender					
Male (reference) Female	0.97 (0.42–2.27)	1.10 (0.54–2.22)	0.96 (0.48–1.93)	1.06 (0.71–1.56)	1.11 (0.65–1.89)
Age at onset					
< 4 years ≥ 4 years (reference)	0.76 (0.35–1.67)	0.50 (0.26–0.95)	0.62 (0.33–1.17)	0.65 (0.45–0.93)	0.45 (0.28–0.73)
Ethnicity					
Caucasian (reference) Asian Others	0.49 (0.11–2.3)	0.79 (0.20–2.46)	1.29 (0.45–3.69)	0.96 (0.55–1.68)	0.89 (0.43–1.84)

*CI* confidence interval, *HR* hazard ratio, *FRNS* frequent relapsing nephrotic syndrome, *OR* odds ratio, *SDNS* steroid-dependent nephrotic syndrome

### Secondary outcomes

**Fig. 3:**
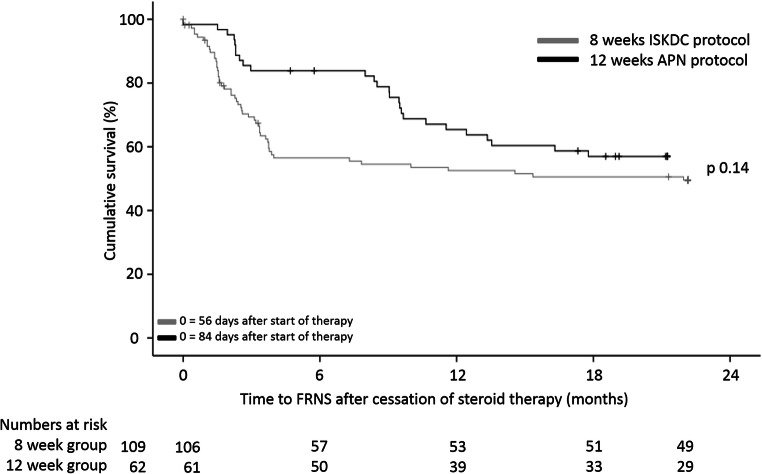
Time to frequent relapsing nephrotic syndrome after cessation of prednisolone therapy. APN, Arbeitsgemeinschaft für Pädiatrische Nephrology; FRNS, frequent relapsing nephrotic syndrome; ISKDC, International Study of Kidney Disease in Children

The proportion of patients developing FRNS within 24 months of follow-up was similar in both groups (Table 2). As shown in Table 3, multivariable logistic regression analysis showed that gender and ethnicity were not significantly associated with the development of FRNS. Children over the age of 4 had a lower chance on developing FRNS (OR = 0.50 [0.26–0.95]). Time to FRNS was numerically better but did not significantly differ between the 8 weeks and 12 weeks treatment groups (Fig. 3). Cox proportional hazards regression analysis suggests children below 4 years of age to have a significantly shorter time to FRNS compared to children above the age of 4 (Table 3). No difference was found regarding the incidence of steroid dependency between the 8 weeks and 12 weeks group (RR = 0.91 [0.65-1.27], Table 3).

At 1 year of follow-up counted from the start of treatment, relapse rates were significantly lower in the 12 weeks treatment group (1.4 relapses per year) compared to the 8 weeks treatment group (2.3 relapses per year) (RRR = 0.64 [0.48–0.84], Table 4). Two years after presentation, the number of relapses was similar in the two treatment groups. However, for the total follow-up duration, relapse rates were 1.2 relapses per year for the 8 weeks treatment group versus 0.8 relapses per year for the 12 weeks group (RRR = 0.66 [0.49–0.90], Table 4). At 1 year of follow-up and at last follow-up, the incidence rate of relapses in children of 4 years and older was significantly lower compared to children below the age of 4 years (Table 4).

**Table 4:** Relapse rates

	Total follow-up* **		1 year of follow-up*		2 years of follow-up*	
Covariate	RR	RRR (95% CI)	RR	RRR (95% CI)	RR	RRR (95% CI)
Treatment group						
8 weeks 12 weeks (reference)	1.2 0.8	0.66 (0.49−0.90)	2.3 1.4	0.64 (0.48−0.84)	1.6 1.4	0.89 (0.67−1.12)
Gender						
Male (reference) Female		1.13 (0.82−1.56)		1.03 (0.78−1.36)		1.12 (0.83−1.52)
Age at onset						
<4 years ≥4years (reference)		0.74 (0.55−0.99)		0.73 (0.56−0.94)		0.83 (0.63−1.09)
Ethnicity						
Caucasian (reference) Asian Other		1.02 (0.82−1.27)		1.04 (0.86−1.26)		1.07 (0.87−1.31)

Abbreviations: *CI* confidence interval; *RR* relapse rate (number of relapses per year); *RRR* relapse rate ratio

*Counted from the start of treatment

**See Supplemental Table 3 for median (IQR) follow-up duration

### Comparison to aggregate trial results

Results of the IPD meta-analysis were compared to the aggregate trial results of the Ehrich trial [17]. The percentage of patients with sustained remission in the 12 weeks treatment group of the Ehrich trial was 49% compared to 23% in the 12 weeks treatment group of the IPD meta-analysis (Table 5). Percentages of sustained remission in both 8 weeks treatment groups were comparable. The percentages of patients having a relapse within 3 or 6 months after the end of continuous therapy are significantly lower in the Ehrich trial (Table 5 and Supplemental Table 4). Similarly, the proportion of patients requiring cytotoxic drug therapy is lower in the Ehrich trial (Table 5, Supplemental Tables 4). The proportion of patients progressing to FRNS is similar (44% versus 32% [8 weeks groups] and 16% versus 18% [12 weeks groups]) (Table 5).

**Table 5:** Comparison of odds ratios of binary outcome measures from the IPD meta-analysis and Ehrich trial, using the endpoints of Ehrich trial

	IPD MA 8 weeks	IPD MA 12 weeks	OR	Ehrich 8 weeks	Ehrich 12 weeks	OR	Difference in odds ratios, *p* Value
Cumulative rate of sustained remission 2 years after initial attack	22/109 20%	14/62 23%	1.15 (0.54–2.46)	7/37 19%	17/34 49%	4.3 (1.48–12.4)	0.049
Relapse within 3 months after end of continuous therapy	65/105 62%	18/62 29%	0.25 (0.13–0.50)	11/37 30%	5/34 15%	0.41 (0.13–1.33)	0.49
Relapse within 6 months after end of continuous therapy	76/105 72%	35/62 56%	0.50 (0.26–0.96)	18/37 49%	8/34 24%	0.33 (0.12–0.90)	0.50
Relapse within 12 months after end of continuous therapy	84/105 80%	44/62 71%	0.61 (0.30–1.27)	24/37 65%	13/34 38%	0.34 (0.13–0.88)	0.33
Steroid toxicity requesting cytotoxic drug therapy	61/109 56%	28/62 45%	0.65 (0.35–1.21)	8/37 22%	5/34 15%	0.63 (0.18–2.14)	0.96
Two or more relapses in any subsequent 6 month period*	52/109 48%	26/62 42%	0.79 (0.42–1.49)	21/37 57%	10/34 29%	0.32 (0.12–0.85)	0.12
FRNS in first 6 months after end of continuous therapy	45/103 44%	10/62 16%	0.25 (0.11–0.54)	12/37 32%	6/34 18%	0.45 (0.15–1.37)	0.40

*IPD MA* individual patient data meta-analysis, *FRNS* frequent relapsing nephrotic syndrome, *IQR* interquartile range, *OR* odds ratio

*Data used for IPD meta-analysis: progression to FRNS within 24 months of follow-up

## Discussion

The results of this IPD meta-analysis suggest that the ISKDC steroid regimen using 8 weeks of steroid treatment for a first episode of steroid-sensitive nephrotic syndrome may not be equally effective as the 12 weeks APN steroid regimen in terms of time to first relapse and relapse rates at 12 months and at total follow-up. The proportion of patients developing FRNS or SDNS where similar in both treatment groups. Interestingly, children below 4 years of age showed a decrease in time to first relapse and time to FRNS and showed higher relapse rates compared to children aged 4 years and older.

To date, only a few small trials were reported in which the 8 weeks and 12 weeks steroid regimens for the first episode of childhood nephrotic syndrome were compared [14–17]. In line with the results of the IPD meta-analysis, Ehrich et al. concluded that the 12 weeks treatment regimen is preferred for the treatment of a first episode of nephrotic syndrome [17]. Overall, a relatively high percentage (49%) of patients in the 12 weeks group having sustained remission after 2 years was reported in the 1993 Ehrich trial, which is not consistent with the results from our analysis and percentages reported in literature [31]. Interestingly, in the 8 weeks group of the 1993 Ehrich trial, the percentage of patients with sustained remission after 2 years was 19%, whereas in the 1988 Ehrich trial this was 41% [17, 30]. Moreover, the proportion of patients requiring cytotoxic drug therapy is low compared to patients included in the IPD meta-analyses. As many aspects of nephrotic syndrome, such as underlying pathology [32], steroid responsiveness [33], and availability of second-line immunosuppressive agents seem to be changing over decades, the apparent difference in outcome may be based on the different era in which the Ehrich study was performed compared with the Teeninga and PREDNOS trials. As previously reported in the Cochrane review of Hahn et al. [13], blinding was not mentioned in the Ehrich trial, which is a major limitation. Norero et al. reported no superiority of the 12 weeks treatment in terms of mean relapse rate per patient in 18 months, number of patients with FRNS, adverse effects, and the number of patients with a relapse at 12 and 18 months of follow-up [14]. Time to first relapse was not reported in this study, and patients progressing to SDNS were excluded from the analysis. Similarly, Paul et al. indicated that prolongation of prednisolone therapy for the initial episode of nephrotic syndrome did not have a beneficial effect on the outcome in the subsequent year [16]. In contrast, Moundekhel et al. concluded that the 12 weeks APN treatment was superior to the 8 weeks ISKDC in preventing relapses in nephrotic syndrome [15]. Of note, the trials of Norero et al., Paul et al., and Moundekhel et al. were reported to have high or unclear risks on several bias items [13]. Importantly, the studies included in this IPD meta-analysis showed a low risk of bias on all bias items of the Cochrane review by Hahn et al. [13].

The purpose of intensification of steroid therapy in nephrotic syndrome is to alter the overall disease course and minimize drug toxicity rather than only delay the first relapse. In light of this, time to first relapse should not be the only parameter to select the preferred treatment of a first episode of nephrotic syndrome and long-term outcome measures should be taken into account. Additionally, our analyses show that relapse rates for the total follow-up duration are significantly higher in the 8 weeks group compared to the 12 weeks group. Interestingly, in the study of Webb et al., no superiority in terms of clinical outcomes of the 16 weeks steroid regimen was found compared to the 8 weeks steroid regimen [10]. In contrast, our IPD meta-analysis shows that time to first relapse after cessation of prednisolone therapy was significantly increased in the 12 weeks treatment group. We hypothesize that the differences in clinical outcomes between the IPD meta-analysis and the trial of Webb et al. may be a consequence of longer daily steroid treatment in the 12 weeks steroid regimen (6 weeks of daily steroids) compared to the 16 weeks steroid regimen (4 weeks of daily steroids). In accordance with our results on FRNS, the recently published Cochrane review of Hahn et al. concluded little or no difference in the number with frequent relapses between the 2 and ≥ 3 months regimens based on studies at low risk of bias [13]. Post-hoc analyses of previous studies have suggested that an early age at onset is a risk marker for a complicated disease course [10, 12]. The results of our study confirm this risk and suggest that children below 4 years of age show a decrease in time to first relapse and time to FRNS, and higher relapse rates, independent from the treatment regimen used. Using cut-off values for age at diagnosis of 3, 5, or 6 years, similar results can be obtained regarding time to FRNS. A subgroup analysis indicated that children below the age of 4 treated according to the 12 weeks regimen did not progress less frequently to FRNS; however it did show a statistically significant increase in time to FRNS. An IPD meta-analysis (by Bagga et al. [personal communication]) including patient data from several large clinical trials is underway to determine whether initial therapy should be prolonged in younger patients with nephrotic syndrome.

Previously, significant practice variation on the treatment of a first episode of nephrotic syndrome has been identified among European centers [8]. To answer the question on the optimal treatment for a first presentation of nephrotic syndrome in Europe, only European trials were included in this IPD meta-analysis. Interestingly, previous studies indicated that Asian children have a higher incidence of nephrotic syndrome with a less complicated disease course compared to European children [20, 21]. This is likely to be associated with differences in genetic factors involved in the pathogenesis of nephrotic syndrome or the response to steroids. Multivariate regression analysis indicated that ethnicity was not significantly associated with any of our outcomes. As a small effect cannot be excluded, we performed a subgroup analysis excluding patients with a non-European descent. We found similar results in time to first relapse, incidence of SDNS and FRNS, relapse rates at 12 months, 24 months, and last follow-up. In this analysis, time to FRNS was significantly shorter in the 12 weeks group (HR 0.43 [0.23–0.78]).

One of the strengths of our study is that a comparison of the two commonly used treatment arms is performed in a large group of patients. The use of IPD offers advantages over analyses using aggregate data extracted from publications. Aggregate data are often presented non-uniformly across studies and more likely to be reported when statistically or clinically significant which may lead to publication bias and selective reporting. Individual-level information enables more flexible and robust analyses than are possible with aggregate study results [18]. The statistical analysis and outcome measures are standardized across studies. Finally, baseline factors can be adjusted for consistently across studies, and IPD meta-analyses may allow detailed exploration of interactions between participant characteristics and treatment effects [34, 35]. Taken together, IPD meta-analyses often provide more detailed and reliable results and a greater depth of understanding than is possible from aggregate data [36]. This IPD meta-analysis also has its limitations. IPD were available for 50% (2/4) of the included trials. In randomized controlled trials, randomization causes a fair distribution of patient variables among the two treatment groups. As we compared two treatment arms from two independent studies, patient variables were not randomly distributed. To overcome this potential problem, baseline characteristics were compared between both groups, and all analyses were adjusted for the covariates to control confounding. The cumulative amount of steroids used during follow-up was not available, nor could we compare data on adverse events and time to SDNS. Finally, we chose to only include European trials in the IPD meta-analysis to prevent interference from ethnicity on outcomes as we aimed to answer the question on the optimal treatment regimen for European children with nephrotic syndrome. This, however, hampers the generalizability of the results, and therefore the results should be interpreted with caution for patients with a non-European descent.

In conclusion, the results of this IPD meta-analysis suggest that the 8 weeks steroid regimen for a first episode of steroid-sensitive nephrotic syndrome may not be equally effective as the 12 weeks steroid regimen. Although less steroids for the first nephrotic syndrome episode would be beneficial in terms of steroid toxicity in the short term, these results suggest patients treated with a 12 weeks steroid regimen may have a lower number of relapses during follow-up. Nevertheless, as the number of patients developing FRNS or SDNS was similar in the two treatment groups, the final conclusion on the preferred treatment for a first episode of childhood nephrotic syndrome remains to be elucidated. To further minimize steroid toxicity alternative strategies may be considered including dose reduction [37] or addition of second-line immunosuppressive drugs for a first manifestation of nephrotic syndrome [38, 39].

## Supplementary information

ESM 1 (PDF 132 kb)

## Data Availability

The datasets analyzed during the current study are available from the corresponding author in consultation with the primary investigators of the trials on reasonable request.

## Abbreviations

APN: Arbeitsgemeinschaft für Pädiatrische Nephrologie
CI: Confidence intervals
FRNS: Frequently relapsing nephrotic syndrome
HR: Hazard ratio
IPD: Individual patient data
ISKDC: International Study of Kidney Disease in Children
KDIGO: Kidney Disease: Improving Global Outcomes
PRISMA: Preferred Reporting Items for Systematic Review and Meta-Analysis
RR: Relative risks
RRR: Relapse rate ratios
SDNS: Steroid-dependent nephrotic syndrome
US: United States
