# Supplementary material for: Steroid treatment for the first episode of childhood nephrotic syndrome: comparison of the 8 and 12 weeks regimen using an individual patient data meta-analysis
Source: Eur J Pediatr. 2021 Mar 28;180(9):2849–59. doi: 10.1007/s00431-021-04035-w (PMC8346453; doi:10.1007/s00431-021-04035-w)
Supplement: Supplementary file 1 — (PDF 132 kb) [file 431_2021_4035_MOESM1_ESM.pdf]

Supplementary Information

Steroid treatment for the first episode of childhood nephrotic syndrome: comparison of the 8 and 12 weeks regimen using an individual patient data meta-analysis

Anne M. Schijvens, MD[1], Nynke Teeninga, MD PhD[1], Eiske M. Dorresteijn, MD[2], Steven Teerenstra[3], Nicholas J. Webb[4,5], MD PhD, Michiel F. Schreuder, MD PhD[1]

[1] Radboud university medical center, Radboud Institute for Molecular Life Sciences, Amalia Children's Hospital, Department of Pediatric Nephrology, Nijmegen, The Netherlands

[2] Erasmus University Medical Center, Sophia Children's Hospital, Department of Pediatric Nephrology, Rotterdam, The Netherlands

[3] Department for Health Evidence, Radboud Institute for Health Sciences, Radboud University Medical Center, Nijmegen, The Netherlands.

[4] Royal Manchester Children's Hospital, Department of Paediatric Nephrology, Manchester, UK.

[5] Manchester Academic Health Science Centre, University of Manchester, Manchester M13 9PL, UK

Anne.schijvens@radboudumc.nl
Journal: European Journal of Pediatrics

Table of contents

**1**	**Supplemental Table 1** Definitions used for FRNS and SDNS
**2**	**Supplemental Table 2** Identified trials on steroid treatment of first presentation of nephrotic syndrome in children
**3**	**Supplemental Table 3** Study characteristics of the included trials
**4**	**Supplemental Table 4** Comparison of 8 and 12 weeks treatment groups of IPD meta-analysis and Ehrich trial
**5**	**Supplementary references**

Supplemental Table 1 Definitions used for FRNS and SDNS

	Definitions		
1	KDIGO	FRNS	Two or more relapses in the first 6 months of initial response, or four or more relapses in any 12-month period
		SDNS	Two or more consecutive relapses during corticosteroid therapy, of within 14 days of ceasing therapy
2	Webb trial	FRNS	Two or more relapses in the first 6 months **following presentation** or four or more relapses in any 12 month period
	Teeninga trial	FRNS	A. Two or more relapses within 6 months **after completing initial treatment**<br>B. Four relapses within any period of 12 months, including relapses during initial treatment<br>(C. FRNS based on a clinical decision that included additional treatment: prednisolone maintenance therapy (>three months) or other immunosuppressive agents)
	Webb trial	SDNS	**Relapse** while receiving corticosteroid treatment or within 14 days of stopping the drug
	Teeninga trial	SDNS	**Two or more consecutive relapses** either during or within 14 days of completion of steroid therapy
3	To enable comparison of trial results	FRNS	Two or more relapses in the first 6 months **following presentation** or four or more relapses in any 12 month period
		SDNS	**Relapse** while receiving corticosteroid treatment or within 14 days of stopping the drug

Abbreviations: FRNS, frequent relapsing nephrotic syndrome; SDNS, steroid dependent nephrotic syndrome.

Supplemental Table 2 Identified trials on steroid treatment of first presentation of nephrotic syndrome in children

	Trial	Short treatment arm	Long treatment arm	Comment	Ref.
1	APN, 1988	60 mg/m^2 daily until protein-free urine, 40mg/m^2 AD until albumin >35 g/l	60mg/m^2 daily for 4 weeks, 40mg/m^2 AD for 4 weeks	Individual patient data unavailable	[S1]
2	Ueda, 1988	60 mg/m2 daily for 4 weeks, 40 mg/m2 on 3/7 consecutive days for 4 weeks	60 mg/m2 daily for 4 weeks, 40 mg/m2 AD for 4 weeks and taper by 10mg/m^2 per month.	Other dosing regimens, non-European trial	[S2]
3	Ehrich, 1993	60 mg/m^2 daily for 4 weeks, 40 mg/m^2 AD for 4 weeks	60 mg/m^2 daily for 6 weeks, 40 mg/m^2 AD for 6 weeks	Individual patient data unavailable	[S3]
4	Ksiazek, 1995	1-2 mg/kg daily for 4 weeks,<br>1 mg/kg AD for 4 weeks	1-2 mg/kg daily for 4 weeks,<br>1 mg/kg AD for 4 weeks and taper by 25% each month for 4 months or taper by 25% each week for 4 weeks	Other dosing regimens	[S4]
5	Norero, 1996	60 mg/m^2 daily for 4 weeks, 40 mg/m^2 AD for 4 weeks	60 mg/m^2 daily for 6 weeks, 40 mg/m^2 AD for 6 weeks	Non-European trial	[S5]
6	Yoshikawa, 1998	2 mg/kg daily for 4 weeks, 1.3 mg/kg AD for 4 weeks	2 mg/kg daily for 4 weeks, 2 mg/kg AD for 8 weeks, 1.5 mg/kg AD for 2 weeks, 0.5 mg/kg AD for 2 weeks	Other dosing regimens, co-intervention with Chinese herb, non-European trial.	[S6]
7	Bagga, 1999	2 mg/kg daily for 4 weeks,<br>1.5 mg/kg AD for 4 weeks	2 mg/kg daily for 4 weeks, 1.5 mg/kg AD for 4 weeks, 1 mg/kg AD for 4 weeks	Non-European trial	[S7]
8	Hiraoka, 2000	40 mg/m^2 daily for 6 weeks, 40 mg/m^2 AD for 6 weeks	60 mg/m^2 daily for 6 weeks, 40 mg/m^2 AD for 6 weeks	Non-European trial	[S8]
9	Sharma, 2000	60 mg/m^2 daily for 6 weeks, 40 mg/m^2 AD for 6 weeks	60 mg/m^2 daily for 6 weeks, 40 mg/m^2 AD for 6 weeks, taper by 10mg/m^2 each month for 3 months	Only reported in abstract form, non-European trial	(Sharma RK 2000, CN-00550434)
10	Satomura, 2001	60 mg/m^2 daily for 4 weeks, 40 mg/m^2 AD for 4 weeks	40 mg/m^2 daily for 4 weeks, 40 mg/m^2 AD for 8 weeks	Only reported in abstract form, non-European trial.	(Satomura K 2001, CN-00447593)
11	Jayantha, 2002	60 mg/m^2 daily for 4 weeks, 40 mg/m^2 AD for 4 weeks	60 mg/m^2 daily for 4 weeks,<br>60 mg/m^2 AD, taper by 10mg/m^2 every 4 weeks	Only reported in abstract form, non-European trial.	(Jayantha UK, 2004, CN-00583710)
12	Hiraoka, 2003	60 mg/m^2 daily for 6 weeks, 40 mg/m^2 AD for 6 weeks	60 mg/m^2 daily for 4 weeks, 60 mg/m^2 AD for 4	Non-European trial	[S9]

			weeks, taper by 10mg/m^2 each month		
13	Pecoraro, 2003	2 mg/kg daily for 4 weeks, 2 mg/kg AD for 4 weeks, taper by 0.25 mg/week	2mg/kg daily of 6 weeks, 2 mg/kg AD for 6 weeks, taper by 0.25 mg/week<br><br>IV methylprednisolone 20mg/kg daily for 3 days, 1 mg/kg/day for 6 weeks, 1 mg/kg AD for 6 weeks, taper by 0.25 mg/week.	Other dosing regimens. Only reported in abstract form.	(Pecoraro C, 2005, CN-00644161)
14	Moundekhel, 2012	60 mg/m^2 daily for 4 weeks, 40 mg/m^2 AD for 4 weeks	60 mg/m^2 daily for 6 weeks, 40 mg/m^2 AD for 6 weeks	Non-European trial	[S10]
15	Mishra, 2012	2 mg/kg daily for 6 weeks, 1.5 mg/kg AD for 6 weeks	2 mg/kg daily for 6 weeks, 1.5 mg/kg AD for 6 weeks, 1 mg/kg for 4 weeks, 0.5 mg/kg AD for 4 weeks	Non-European trial	[S11]
16	Teeninga, 2013	60 mg/m^2 daily for 6 weeks, 40 mg/m^2 AD for 6 weeks	60 mg/m^2 daily for 10 days, 50 mg/m^2 daily for 6 weeks, 40 mg/m^2 AD until end week 10, 30 mg/m^2 AD until end week 14, 10 mg/m^2 AD until end week 24	Short treatment arm included in IPD meta-analysis	[S12]
17	Paul, 2014	60 mg/m^2 daily for 4 weeks, 40 mg/m^2 AD for 4 weeks	60 mg/m^2 daily for 6 weeks, 40 mg/m^2 AD for 6 weeks	Non-European trial	[S13]
18	Yoshikawa, 2015	60 mg/m^2 daily (3 divided doses) for 4 weeks, 40 mg/m^2 AD for 4 weeks	60 mg/m^2 daily (3 divided doses) for 4 weeks, 60 mg/m^2 AD for 4 weeks, 45 mg/m^2 AD for 4 weeks, 15 mg/m^2 AD for 4 weeks.	3 divided doses daily, non-European trial.	[S14]
19	Sinha, 2015	2 mg/kg daily for 6 weeks, 1.5 mg/kg AD for 6 weeks	2 mg/kg daily for 6 weeks, 1.5 mg/kg AD for 6 weeks, 1 mg/kg AD for 4 weeks, 0.5 mg/kg AD for 4 weeks, 0.25 mg/kg AD for 4 weeks,	Non-European trial	[S15]
20	Al Talhi, 2018	60 mg/m^2 daily for 6 weeks, 40 mg/m^2 AD for 6 weeks	60 mg/m^2 daily for 4 weeks, 40 mg/m^2 AD for 8 weeks, 30 mg/m^2 AD for 8 weeks, 20 mg/m^2 AD for 8 weeks	Non-European trial	[S16]
21	Webb, 2019	60 mg/m^2 daily for 4 weeks, 40 mg/m^2 AD for 4 weeks	60 mg/m^2 daily for 4 weeks, followed by 12 weeks of AD prednisolone treatment, starting at 60 mg/m2 and tapering by 10 mg/m2 every two weeks.	Short treatment arm included in IPD meta-analysis	[S17]

AD, alternate days; IPD, individual patient data

Supplemental Table 3 Study characteristics of the included trials

	**Webb 2019 (S17)**	**Teeninga 2013 (S12)**	**Ehrich 1993 (S3)**
**Information**	Individual patient data	Individual patient data	Aggregate trial results
**Treatment arm 1**	Extended course group:<br>prednisolone 60mg/m^2 daily for 4 weeks, followed by 12 weeks of AD prednisolone treatment, starting at 60mg/m^2 and tapering by 10 mg/m^2 every two weeks.	Extended course group:<br>24 week schedule using the same cumulative prednisolone dose as standard course group.<br><br>60 mg/m^2 daily until remission, 50 mg/m^2 until 6 weeks after presentation, 40 mg/m^2 AD for 4 weeks, 20mg/m^2 AD for 4 weeks, 10mg/m^2 AD for 10 weeks.	Extended course group:<br>prednisolone 60mg/m^2 daily for 6 weeks, followed by 40mg/m^2 on AD for 6 weeks.
**Treatment arm 2**	Standard course group:<br>prednisolone 60mg/m^2 daily for 4 weeks, followed by 40mg/m^2 on AD for 4 weeks.	Standard course group:<br>prednisolone 60mg/m^2 daily for 6 weeks, followed by 40mg/m^2 on AD for 6 weeks.	Standard course group:<br>prednisolone 60mg/m^2 daily for 4 weeks, followed by 40mg/m^2 on AD for 4 weeks.
**Sample size**	237	126	71
**Age at diagnosis**<br>**mean years (SD)**<br>**median years (IQR)**<br>**median years (range)**	 4.9 (3.1)	 <br>4.2 (3.2-6.2)	 <br><br>3.9 (1.5-8) (extended course)<br>4.4 (1.5-14) (standard course)
**Sex (%)**	65% male	68% male	ND
**Reported outcomes**	Time to first relapse, relapse rate, incidence of FRNS, SDNS, use of alternative immunosuppressive treatment, rates of adverse events, behavioral change, QALY, cost-effectiveness	FRNS, cumulative incidences of a first relapse, steroid dependence, the number of relapses per patient per year, adverse effects	Cumulative rate of sustained remission, proportion of patients relapsing after 3, 6, and 12 months of follow-up, mean relapse rate at 3,6, and 12 months, mediation duration of remission, FRNS, adverse effects, mean cumulative prednisone dosage, cytotoxic drug therapy.
**Median duration of follow-up months (IQR)**<br><br><br>**Mean duration of follow-up months (IQR)**	Standard course group<br>37 (30-48)	Extended course group<br>47 (37-60)<br>Standard course group<br>46 (32-60)	 <br><br><br>Extended course group<br>20 (ND)

			Standard course group<br>22 (ND)
**Trial registration**	ISRCTN16645249, EudraCT 2010-022489-29, approved by the North West 7 Research Ethics Committee, Manchester, United Kingdom (10/H1008/122). The trial was carried out under a clinical trial authorisation in accordance with the Medicines for Human Use (Clinical Trials) Regulations (21761/0255/001-0001) and conducted in accordance with the Declaration of Helsinki.	NTR255, approved by the medical ethics committee of the Erasmus University Medical Center in Rotterdam, The Netherlands.	

Supplemental Table 4 Comparison of 8 and 12 weeks treatment groups of IPD meta-analysis and Ehrich trial

	IPD MA 8 weeks	Ehrich 8 weeks	p-value	IPD MA 12 weeks	Ehrich 12 weeks	p-value
**Cumulative rate of sustained remission 2 years after initial attack**	22/109 20%	7/37 19%	0.86	14/62 23%	17/34 49%	< 0.01
**Relapse within 3 months after end of continuous therapy**	65/105 62%	11/37 30%	< 0.01	18/62 29%	5/34 15%	0.02
**Relapse within 6 months after end of continuous therapy**	76/105 72%	18/37 49%	< 0.01	35/62 56%	8/34 24%	<0.01
**Relapse within 12 months after end of continuous therapy**	84/105 80%	24/37 65%	0.02	44/62 71%	13/34 38%	<0.01
**Steroid toxicity requesting cytotoxic drug therapy**	56%	8/37 22%	<0.01	28/62 45%	5/34 15%	<0.01
**Two or more relapses in any subsequent 6 month period***	52/109 48%	21/37 57%	0.20	26/62 42%	10/34 29%	0.06
**FRNS strict in first 6 months after end of continuous therapy**	45/103 44%	12/37 32%	0.11	10/62 16%	6/34 18%	0.71

Abbreviations: IPD MA, individual patient data meta-analysis; FRNS, frequent relapsing nephrotic syndrome; IQR, interquartile range
*Data used for IPD meta-analysis: progression to FRNS within 24 months of follow-up

Supplementary references

S1. (1988) Short versus standard prednisone therapy for initial treatment of idiopathic nephrotic syndrome in children. Arbeitsgemeinschaft fur Padiatrische Nephrologie. Lancet 1:380-383.

S2. Ueda N, Chihara M, Kawaguchi S, Niinomi Y, Nonoda T, Matsumoto J, Ohnishi M, Yasaki T (1988) Intermittent versus long-term tapering prednisolone for initial therapy in children with idiopathic nephrotic syndrome. The Journal of pediatrics 112:122-126.

S3. Ehrich JH, Brodehl J (1993) Long versus standard prednisone therapy for initial treatment of idiopathic nephrotic syndrome in children. Arbeitsgemeinschaft fur Padiatrische Nephrologie. European journal of pediatrics 152:357-361.

S4. Ksiazek J, Wyszynska T (1995) Short versus long initial prednisone treatment in steroid-sensitive nephrotic syndrome in children. Acta Paediatrica, International Journal of Paediatrics 84:889-893.

S5. Norero C, Delucchi A, Lagos E, Rosati P (1996) [Initial therapy of primary nephrotic syndrome in children: evaluation in a period of 18 months of two prednisone treatment schedules. Chilean Co-operative Group of Study of Nephrotic Syndrome in Children]. Rev Med Chil 124:567-572.

S6. Yoshikawa N, Ito H, Takekoshi Y, Honda M, Awazu M, Iijima K, Nakamura H, Seino Y, Takeda N, Hattori S, Matsuda I (1998) Standard versus long-term prednisolone with Sairei-to for initial therapy in childhood steroid-responsive nephrotic syndrome: A prospective controlled study. [Japanese]. Japanese Journal of Nephrology 40:587-590.

S7. Bagga A, Hari P, Srivastava RN (1999) Prolonged versus standard prednisolone therapy for initial episode of nephrotic syndrome. Pediatric nephrology 13:824-827.

S8. Hiraoka M, Tsukahara H, Haruki S, Hayashi S, Takeda N, Miyagawa K, Okuhara K, Suehiro F, Ohshima Y, Mayumi M (2000) Older boys benefit from higher initial prednisolone therapy for nephrotic syndrome. The West Japan Cooperative Study of Kidney Disease in Children. Kidney international 58:1247-1252.

S9. Hiraoka M, Tsukahara H, Matsubara K, Tsurusawa M, Takeda N, Haruki S, Hayashi S, Ohta K, Momoi T, Ohshima Y, Suganuma N, Mayumi M (2003) A randomized study of two long-course prednisolone regimens for nephrotic syndrome in children. American Journal of Kidney Diseases 41:1155-1162.

S10. Moundekhel S, G K, U A, Email Moundekhel S, shamyl, m, khn@gmail, com (2012) Management of nephrotic syndrome: ISKDC versus APN. Pak J Med Health Sci 6:212-215.

S11. Mishra OP, Thakur N, Mishra RN, Prasad R (2012) Prolonged versus standard prednisolone therapy for initial episode of idiopathic nephrotic syndrome. Journal of nephrology 25:394-400.

S12. Teeninga N, Kist-van Holthe JE, van Rijswijk N, de Mos NI, Hop WC, Wetzels JF, van der Heijden AJ, Nauta J (2013) Extending prednisolone treatment does not reduce relapses in childhood nephrotic syndrome. Journal of the American Society of Nephrology : JASN 24:149-159.

S13. Barbour S, Beaulieu M, Gill J, Espino-Hernandez G, Reich HN, Levin A (2014) The need for improved uptake of the KDIGO glomerulonephritis guidelines into clinical practice in Canada: a survey of nephrologists. Clinical kidney journal 7:538-545.

S14. Yoshikawa N, Nakanishi K, Sako M, Oba MS, Mori R, Ota E, Ishikura K, Hataya H, Honda M, Ito S, Shima Y, Kaito H, Nozu K, Nakamura H, Igarashi T, Ohashi Y, Iijima K, Japanese Study Group of Kidney Disease in C (2015) A multicenter randomized trial indicates initial prednisolone treatment for childhood nephrotic syndrome for two months is not inferior to six-month treatment. Kidney international 87:225-232.

S15. Sinha A, Saha A, Kumar M, Sharma S, Afzal K, Mehta A, Kalaivani M, Hari P, Bagga A (2015) Extending initial prednisolone treatment in a randomized control trial from 3 to 6 months did not significantly influence the course of illness in children with steroid-sensitive nephrotic syndrome. Kidney international 87:217-224.

S16. Al Talhi A, Al Saran K, Osman ET, Al Shatri A, Osman M, Mirza K (2018) A randomized study on a 3-month versus a 7-month prednisolone regimen for the initial episode of childhood idiopathic nephrotic syndrome at a large Saudi center. Int J Pediatr Adolesc Med 5:18-23.

S17. Webb NJA, Woolley RL, Lambe T, Frew E, Brettell EA, Barsoum EN, Trompeter RS, Cummins C, Deeks JJ, Wheatley K, Ives NJ, Group PC (2019) Long term tapering versus standard prednisolone treatment for first episode of childhood nephrotic syndrome: phase III randomised controlled trial and economic evaluation. Bmj 365:l1800.
